# Social modulation of object-directed but not image-directed actions

**DOI:** 10.1371/journal.pone.0205830

**Published:** 2018-10-23

**Authors:** Jill A. Dosso, Alan Kingstone

**Affiliations:** Department of Psychology, University of British Columbia, Vancouver, British Columbia, Canada; Nanjing University, CHINA

## Abstract

There has recently been an increased research focus on the influence of social factors on human cognition, attention, and action. While this represents an important step towards an ecologically valid description of real-world behaviour, this work has primarily examined dyads interacting with virtual stimuli i.e. on-screen images of objects. Though differences between actions to images and real items are known, their relative sensitivity to social factors is largely unknown. We argue that because images and real items elicit different neural representations, patterns of attention, and hand actions, a direct comparison between the magnitude of social effects while interacting with images and real objects is demanded. We examined patterns of reaching as individuals performed a shape-matching game. Images and real objects were used as stimuli, and social context was manipulated via the proximity of an observer. We found that social context interacted with stimulus type to modulate behaviour. Specifically, there was a delay in reaching for distant objects when a participant was facing another individual but this social effect only occurred when the stimuli were real objects. Our data suggest that even when images and real objects are arranged to share the affordance of reachability, they differ in their sensitivity to social influences. Therefore, the measurement of social effects using on-screen stimuli may poorly predict the social effects of actions directed towards real objects. Accordingly, generalizations between these two domains should be treated with caution.

## Introduction

The role of social context on human cognition, attention, and action has recently experienced a surge in research. To a large extent this work involves testing dyads on computer-based tasks [1–3]. While introducing a social component (i.e., dyads) into the lab is an excellent way to enhance the real-world dimension of lab-based investigations, the emphasis on lab-based experimentation does keep one foot firmly outside natural environments. Of course, this would not be an issue if images on a screen were processed in the same manner as real objects. Thus, the present work aims to investigate whether object- and image-directed actions are equally sensitive to social context.

Brain imaging research indicates that, during passive viewing, images are processed in a manner that is similar but not identical to real items [4,5]. Neural recordings with animals also support this distinction [6,7]. Real objects and images differ in the amount of attention they elicit, as well as their desirability, recognisability, memorability, and patterns of use [8–15]. Grasping actions directed towards images differ from object-directed grasps in the unfolding of hand shaping over time [16]. In addition, image- but not object-directed actions are influenced by irrelevant stimulus information, suggesting that these actions are partially mediated by different neural circuitry [17–19]. The lack of haptic feedback about absolute object size seems to be an important driver of these differences [20]. Building on this, fMRI work finds that neural activity in the left anterior intraparietal sulcus (aIPS) differentiates 3D- and 2D-directed grasps during action planning, as well as reaching and grasping as discrete actions [21]. Because touchscreen images are often manipulated using pointing, rather than grasping actions, the different neural circuitry underlying these two actions is also relevant to the present work. Specifically, according to Dual Visuomotor Channel Theory, reach-to-grasp actions rely on a dorso-lateral visual network involving the inferior parietal lobe, while reach-to-point actions activate a dorso-medial visual network involving the superior parietal lobe [22,23]. However, even pictures of objects are capable of priming action-related information in some cases [24,25], so the exact circumstances in which images can and cannot stand-in for real objects have not yet been completely defined. Furthermore, the relationship between these actions, their underlying neural circuitry, and their sensitivity to the larger social context in which they are performed is not well understood.

In the present work, we employ an interactive surface to place on-screen images into a common space with real objects in order to compare the impact of a social manipulation on actions directed towards each. The literature on touchscreen use hints that social behaviour may be different on touchscreens than in other contexts. For example, social norm violations that do not occur when handling real items, such as taking items out of another person’s hands, fighting over items, rotating items to favour their egocentric viewpoint over their partner’s viewpoint, or competing for control over a particular location, are sometimes observed on virtual tabletops [26,27]. However, direct comparisons of specifically social behaviour across real objects and images are lacking.

There is evidence that social context can play a role in shaping object-directed actions. For example, social factors shape action kinematics in target- and time-constrained reach-to-grasp tasks [28–33] and observers are often sensitive to social information contained in action [34–37]. Much less work has examined social influences on reach-to-point or reach-to-touch actions (but see [38]). As mentioned, these two types of actions may be governed by different neural circuitry [22,23]. Since the majority of research has focused on social influences on reach-to-grasp actions directed towards real objects, it is unknown whether similar social influences can be seen when image- rather than object-directed actions are assessed.

Therefore, the present study aims to compare real object- and image-directed actions in their sensitivity to a social manipulation. This comparison provides important context for the interpretation of work which uses virtual stimuli as stand-ins for real objects in studies of individual and group behaviour. We compare actions when participants face a peer versus one placed at distance. Facing a stranger has been shown to change the representation of intervening objects and space [39–41], thus we hypothesize that it may influence stimulus-directed actions as well.

It is vital to note at the outset of this research investigation that real objects and images cannot be experimentally matched across all dimensions. Indeed, if they were perfectly matched they would be, in essence, indistinguishable. Thus, we expect that differences will remain in basic performance measures across real-object and image conditions, despite both conditions taking place in a common reachable space. Indeed, such differences have been documented in the touchscreen literature on complex tasks [15]. However, these differences are not the emphasis of the present work. Rather, our core question is whether performance with real objects and images will interact with social changes derived from manipulating the seating arrangement of participants. In interpreting our results, we focus on two possible scenarios. If social effects are consistent across real objects and images (e.g. facing another individual makes participants more error-prone than the offset seating condition for both real items and images) then one can conclude that images can be taken as a reasonable stand-in for real items when measuring social effects. Alternatively, if social effects interact with stimulus type to modulate behaviour (e.g. facing another individual makes participants more error-prone only when images are used) then one can conclude that real items and images are not interchangeable for the measurement of this type of social effect. Identifying the nature of such differences between stimulus types will be important for future researchers hoping to generalize data involving social phenomena.

## Materials and methods

Ethics approval for this study was obtained from the University of British Columbia’s Behavioural Research Ethics Board (BREB). Written, informed consent was obtained from participants. Participants took part for course credit or were paid $5. The children’s game Concentration has been used in tabletop display research [42,43], so we evaluated participants’ performance on a variant of this task. Pairs of participants were pseudo-randomly assigned into the Real Object or Image stimulus-type groups based on equipment availability at the time of scheduling, and were randomly assigned to Facing or Offset seating conditions within their assigned stimulus type. Testing was conducted with a target sample size of 40 participants per group (i.e. each unique combination of observer seating position and stimulus type). Slightly more participants than this were tested in anticipation of the need to make exclusions. Each group was composed of unique, naïve subjects as participants were not permitted to participate in more than one condition.

### Real object group

Ninety-two participants took part, with nine excluded due to technical issues (*n* = 3), reporting below-normal and uncorrected vision (*n* = 2), reporting awareness of recording equipment or aims of the study (*n* = 2), or failing to comply with task instructions (*n* = 2). The final sample were classified as right-handed (*n* = 73), ambidextrous (*n* = 7), or left-handed (*n* = 3) [44]. Participant self-reported sex was female (*n* = 57) and male (*n* = 26). Mean age was 21.4 years (SD = 4.4; range 17–58). Note that the BREB considers 17-year-old university students to be adults for the purpose of low-risk research such as this. Participants’ self-reported ethnicity was Asian (n = 53), Caucasian/White (*n* = 17), or other/unreported (*n* = 13). Forty-six participants were in the Facing condition (29 female), and thirty-seven were in the Offset condition (28 female). The gender of pairs was uncontrolled.

**Fig 1:**
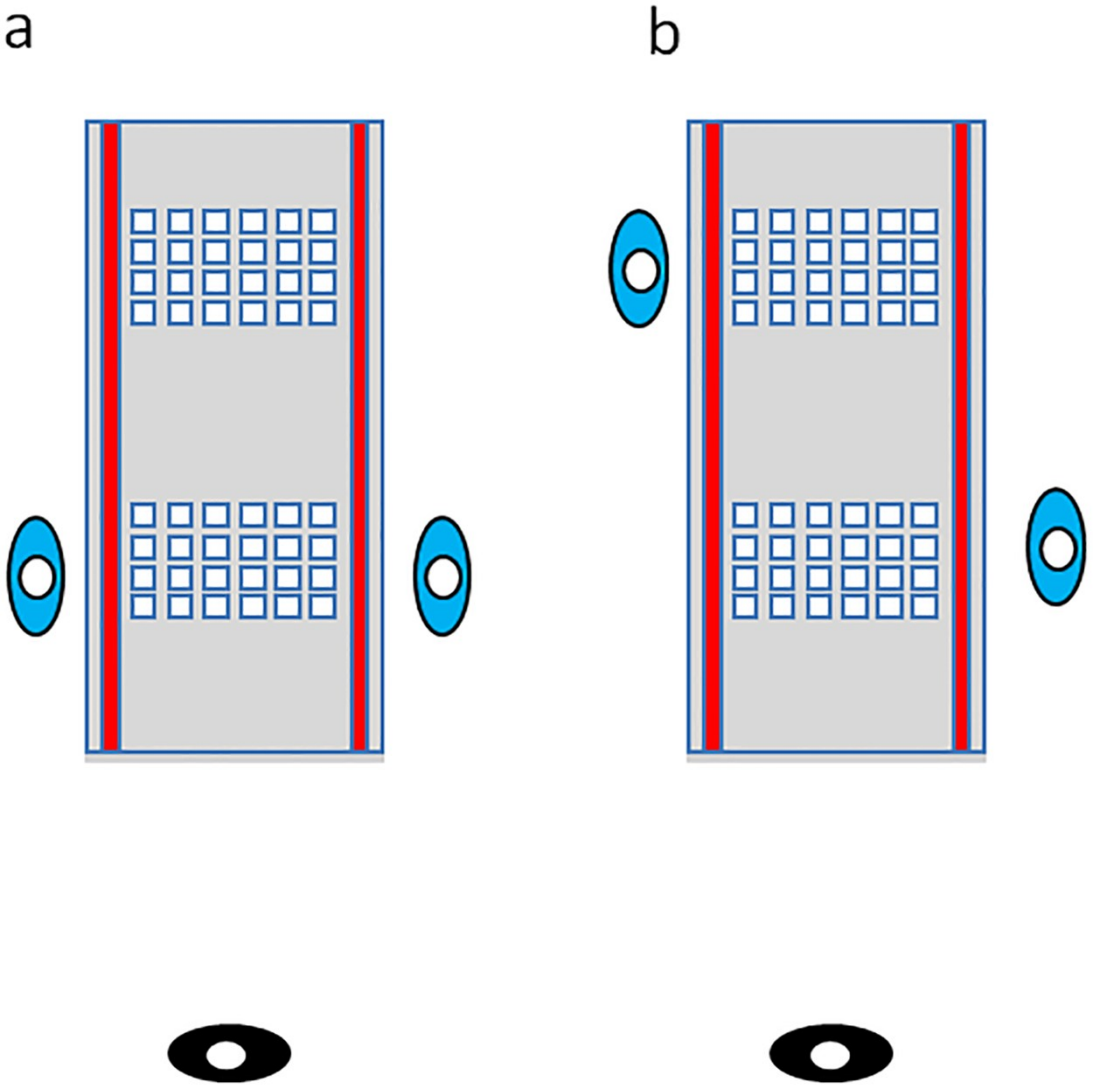
a) Facing and b) Offset seating arrangements used in the real object group. Participants (blue) were seated in front of test array(s). The experimenter (black) observed from a distance of one metre. Not drawn to scale.

On a long table (89 x 74.5 cm), small wooden tiles (5 cm square) were laid out in arrays (37 x 58.5 cm) of 24 items: 4 columns and 6 rows (Fig 1). The experimenter was seated one metre away. A red line was located in front of each participant. Participants were randomly assigned to one of two conditions: Facing or Offset. In the Facing condition, participants were seated face-to-face with 24 tiles face down between them (Fig 1a). The other array location was covered. In the Offset condition, one participant was seated in front of each array, with one participant per side of the table (the total diagonal distance between participants was approximately 115 cm; Fig 1b). Between pairs, seating locations were counterbalanced.

Tiles featured cartoon animals and were placed face down. Participants were instructed to reveal items two at a time. If a matching pair was found, the tiles were left face-up. If a non-matching pair was found, participants were instructed to re-conceal both tiles before continuing. The task continued until all tiles were matched. Participants were also instructed to touch the red line between each reach.

The non-playing participant was instructed to keep their hands on their own line throughout testing. Participant 1 performed the task in its entirety while being passively observed by Participant 2. Then, tiles were reshuffled by the experimenter before Participant 2 was tested with Participant 1 acting as the observer. Participants were instructed to play in a way that minimized the total number of moves used. Minimizing move number, not time, was emphasized. The experimenter reported the number of moves after both participants had performed the task. Participants then completed a short questionnaire about the session as well as a handedness questionnaire [44]. Sessions were inconspicuously video-recorded (at 30 frames per second) to enable later coding of reaching sequence using a laptop placed on a desk to the side of the experimental table. While the laptop camera was on to record participants’ movements, the laptop screen was dark and all lights were off to avoid drawing participants’ attention.

Reaching sequence and time taken from each line touch to each subsequent object touch (“turn length”) was hand-coded from video recordings by two trained coders. Specifically, for each reach to reveal an item the following information was coded: (1) Move number: each turn examining a potential pairs was considered to involve two moves (i.e., flipping over two tiles was counted as two moves); (2) Target distance in terms of row: row 1 to row 6, based on the object’s distance from the participant; (3) Turn length: the time taken from a line touch to the subsequent object touch. Note that this measurement does not include the time taken to rotate and reveal the object’s identity. Coder 1 coded 32 videos alone, coder 2 coded 36 videos alone, and 15 videos were coded independently by both coders. Coding reliability for turn duration was high, Cronbach’s ɑ = 0.97. Agreement for the row of each reach was 99.7%. Coders were blind to the goals of the study.

### Image group

Eighty-two different participants were recruited as above. Two were excluded for failing to comply with task instructions. The sample was classified as right-handed (*n* = 62), ambidextrous (*n* = 7), or left-handed (*n* = 11). Participants’ self-reported sex was female (*n* = 64) and male (*n* = 16). The age range was 17–55 years; mean age was 21.2 years (*SD* = 5.1). Self-reported ethnicity was Asian (*n* = 55), Caucasian (*n* = 15), Multiethnic (*n* = 5), Middle Eastern (*n* = 2), Hispanic (*n* = 1), and unreported (*n* = 2). Forty participated in each of the Facing and Offset conditions (31 and 33 female, respectively).

**Fig 2:**
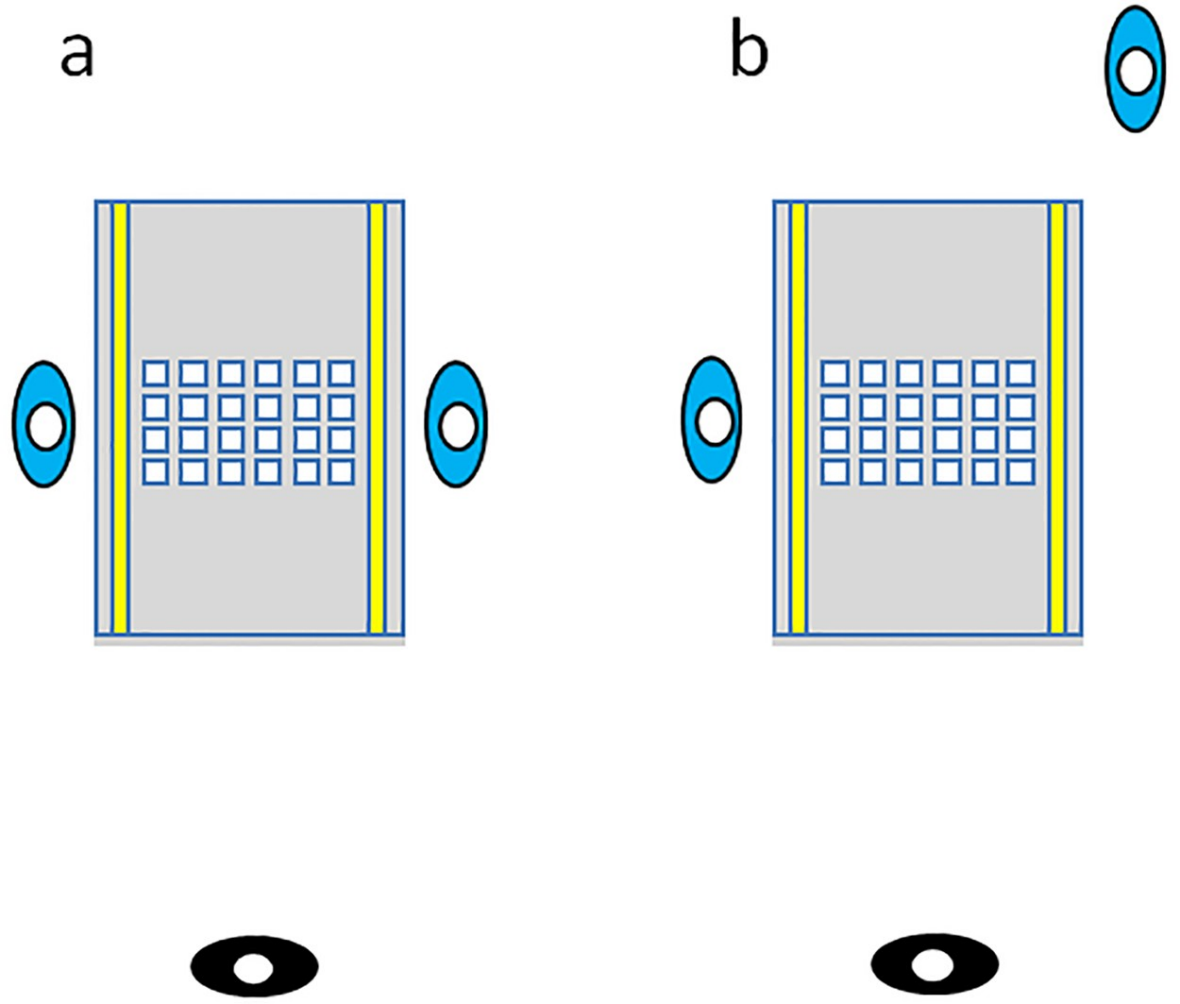
a) Facing and b) Offset seating arrangements used in the image group. Participants (blue) were seated in front of test array(s). The experimenter (black) observed from a distance of one metre. Not drawn to scale.

Participants touched to turn the images on a large horizontal touchscreen (64 cm wide x 105 cm long; Fig 2). As in the Real Object task, participants touched a line in front of themselves between each subsequent touch to an image. The task was programmed using PsychoPy [45], and timing data for each object and line touch were automatically recorded, so no hand-coding was performed. Colourful images of animals on white backgrounds (5 cm square) were taken from the Bank of Standardized Stimuli [46]. Participants were either seated facing one another across the width of the table, or the non-playing participant was seated with a leftward or rightward displacement of one metre (thus, the total diagonal distance between them was 119 cm). Note that a 10.5 cm difference in table widths meant that participants in the Image condition could sit slightly closer to one another, possibly increasing the chance of producing a social effect in the Image condition [47]. The experimenter was again seated one metre away. The non-playing participant was instructed to place their hands on the edge of the touchscreen in the Facing condition, or to keep their hands visible in a similar palm-down posture in their lap in the Offset condition. As above, each participant completed the task in its entirety while being passively observed by the other participant. Between testing of the first and second participant, the layout of the images was digitally reshuffled. After testing, both participants completed a short questionnaire (see Results).

## Results

The participant questionnaire contained 4 ratings (as well as the reported demographic information): overall interest in the task, perception of task difficulty, feelings of competitiveness, and extent to which they liked their partner (all rated on a 1–5 scale). Only ratings of task difficulty differed significantly between groups. Specifically, the object groups found the task harder than the image groups (*F*(1, 162) = 9.9, *p* = .002, η_p_^2^ = .06), and for the object group only, the task was rated as more difficult for the offset as compared to the facing group (*t*(81) = -2.4, *p* = .02).

### Timing

**Fig 3:**
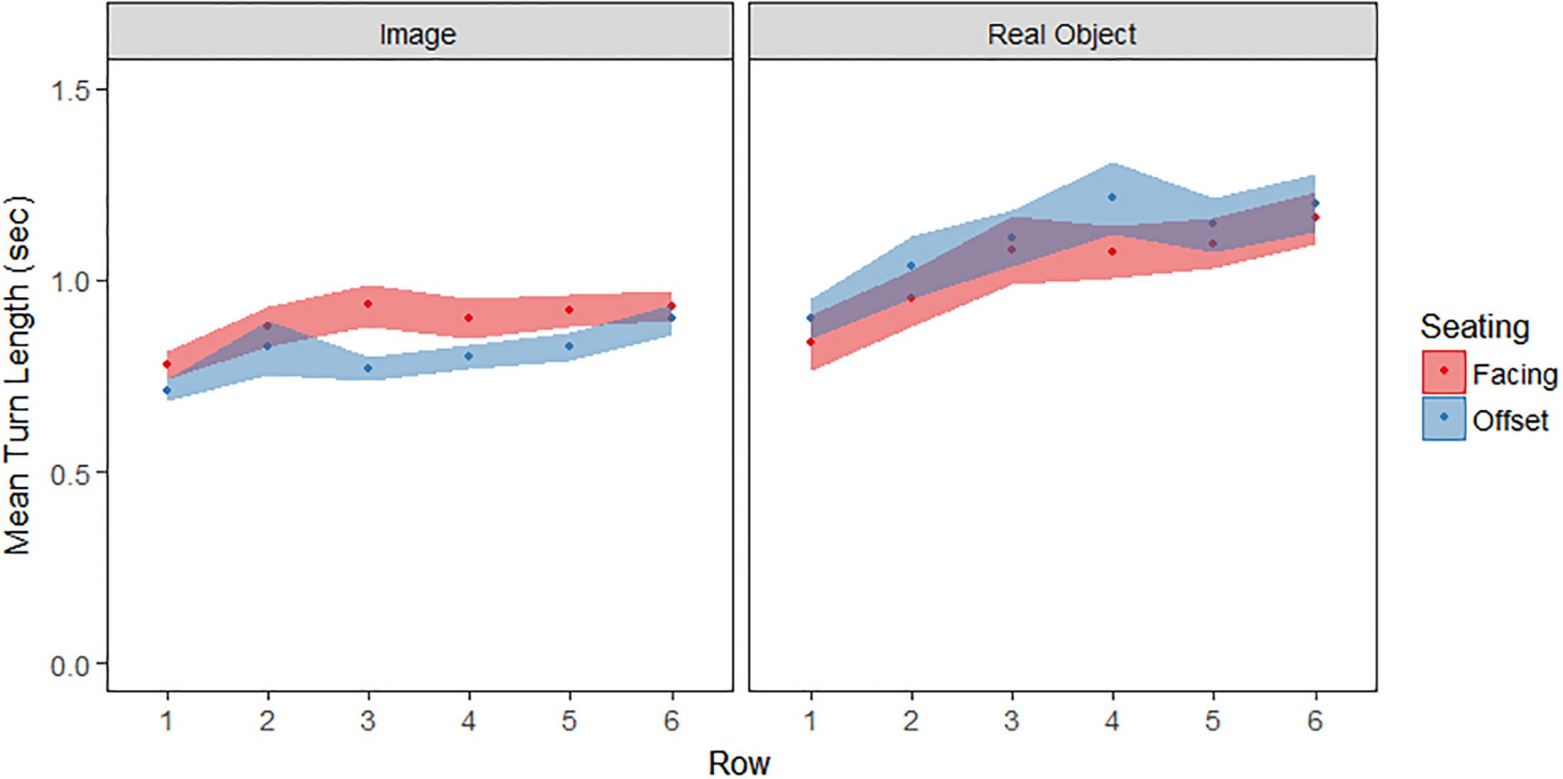
Mean turn duration in seconds (*M*±*SE*) for moves performed.

We defined “turn length” as the time taken from a line touch to the subsequent image touch. This controlled for the extra object handling required when manipulating real objects. Data points more than 2SD above the average for each participant and each row were excluded to control for off-task factors (e.g. mind wandering). Data were also excluded from each participant’s first reach of the testing session, since this often coincided with a last clarification of the study procedure between participant and experimenter (but note that the inclusion of these trials does not change the observed pattern of results, and the effects that were or were not significant). In all, 12.1% of the data was excluded for this analysis. A repeated-measures ANOVA on turn length (see Fig 3) with row (6: rows 1–6) as a within-subjects variable and seating (2: facing, offset) and stimulus type (2: object, image) as between-subjects variables was conducted. This returned a main effect of row (*F*(5, 795) = 25.2, *p* < .001, η_p_^2^ = .14); turns took longer when more distant objects were selected and a main effect of stimulus type (*F*(1, 159) = 16.3, *p* < .001, η_p_^2^ = .09). Participants engaging with real objects rather than images took longer turns. No other effects were observed (all *p*>.15, η_p_^2^ < .02).

### Errors

**Fig 4:**
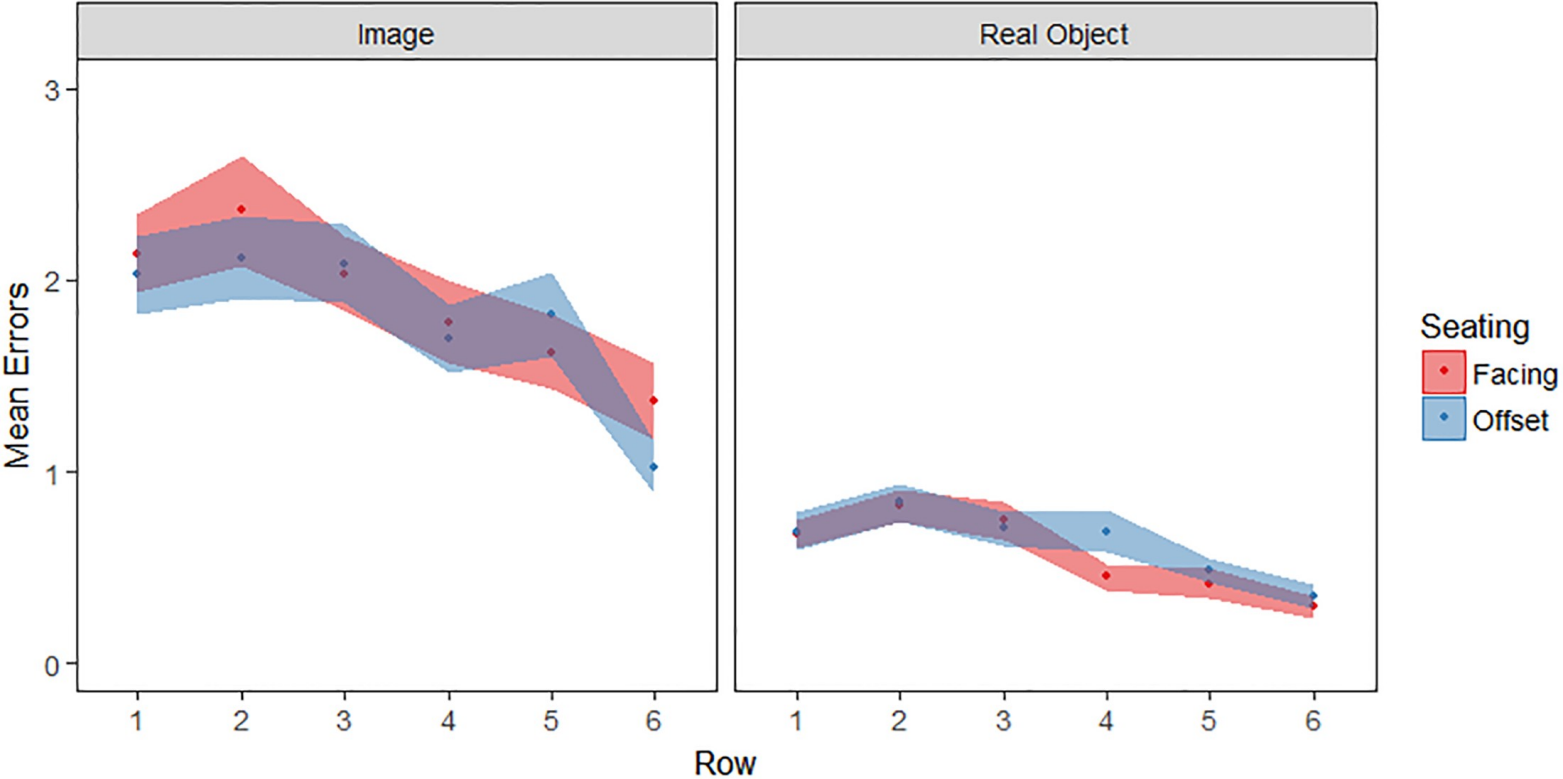
Location of errors (*M*±*SE*).

Errors were defined as repeated flips of a previously viewed item that nevertheless did not lead to a successful match (Fig 4). A repeated-measures ANOVA was conducted with the same factors as before. There was a main effect of stimulus type (*F*(1, 159) = 130.5, *p* < .001, η_p_^2^ = .45); with images producing more errors than real objects. We obtained a main effect of row on erroneous flips (*F*(5, 795) = 20.9, *p* < .001, η_p_^2^ = .12); fewer errors were made in more distant rows than in nearby rows. Row and stimulus type also interacted (*F*(5, 795) = 2.7, *p* = .02, η_p_^2^ = .02) with a greater decline in errors across rows for images. No other effects were significant (all *p*≥.50, all η_p_^2^ < .01).

### Efficiency scores

**Fig 5:**
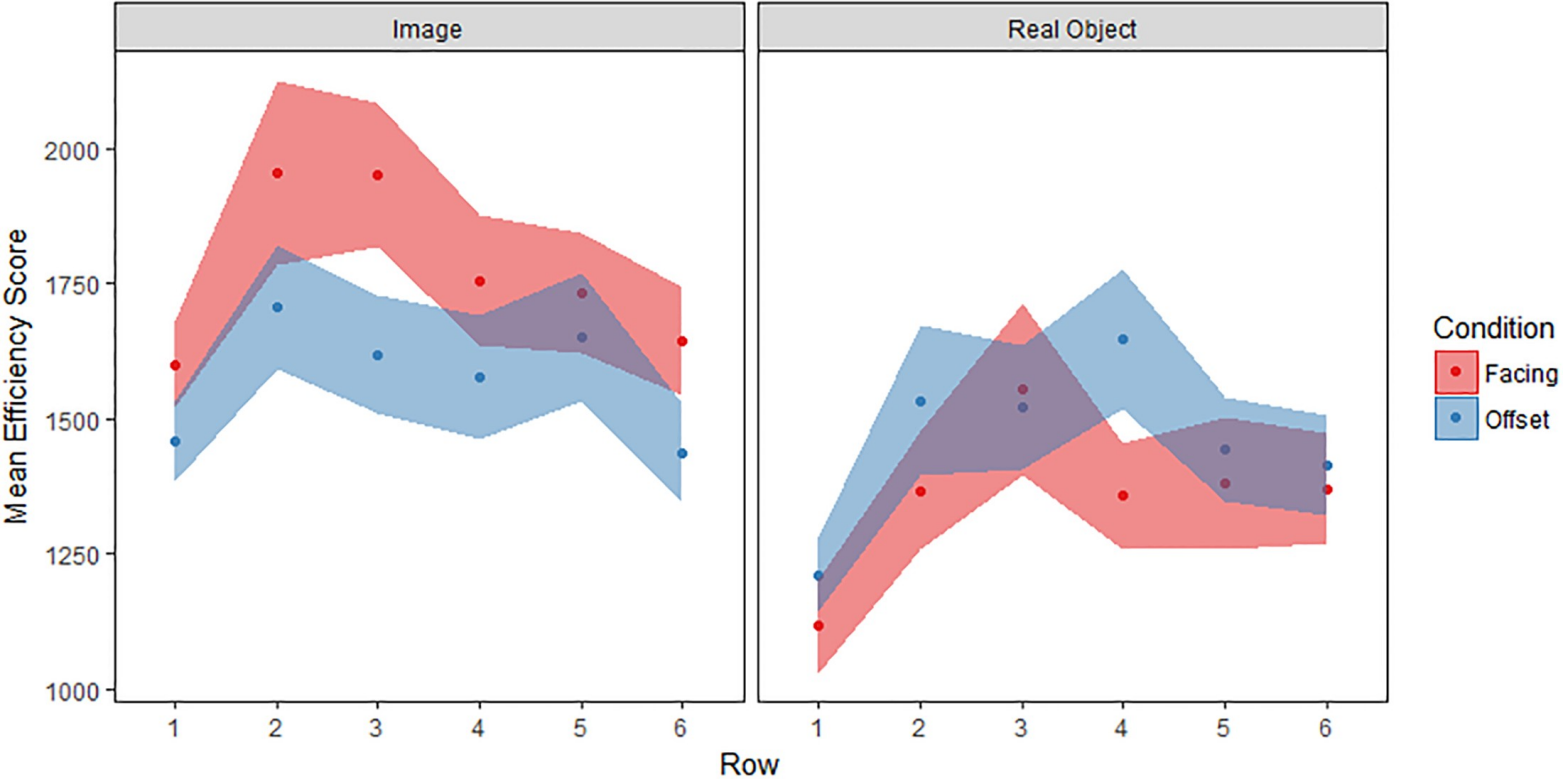
Efficiency scores (M±SE).

To address the possibility of speed-accuracy trade-offs in these data, we calculated efficiency scores (Fig 5) as mean turn length per participant per row, divided by the proportion of reaches that were not errors (i.e. where the item in question had either never been seen before or was successfully matched). A repeated-measures ANOVA was conducted on these efficiency scores, with row (rows 1–6) as a within-subjects variable and stimulus type (object, image) and seating (facing, offset) as between-subjects variables. This analysis produced a main effect of row (*F*(5, 795) = 9.5, *p* < .001, η_p_^2^ = .06) and a main effect of stimulus type (*F*(1, 159) = 2.8, *p* = .02, η_p_^2^ = .05). Thus, we find that efficiency scores varied across array locations (with moves into the first row being most efficient) with object-directed actions having smaller, that is, more efficient scores than image-directed actions across the array. In other words, the two stimulus types differed in their action timing and error rates, and this was not explained by a speed-accuracy trade-off. No other main effects or interactions reached significance (all *p*>.09, all η_p_^2^ < .02) including the social manipulation: seating position.

### Reaching sequence

In order to quantify participants’ exploration of the array, the move number and target distance (row 1 to row 6) were analysed. Each participant provided 6 data points for this analysis: the move number on which they first contacted an item in Row 1, Row 2, etc. This measure provides an index of how rapidly participants investigate the whole array versus repeatedly examine tiles in the same region of space. For example, an actual participant in this data set who acted fairly systematically in moving from interacting with nearby objects to more distant objects had a data series of 1, 5, 9, 15, 39, 53 for Rows 1–6, i.e. on move 1 the first row was contacted for the first time, on move 5 the second row was contacted for the first time, and so forth with the most distant sixth row not contacted until move 53. By contrast, another participant reached into more distant rows early in their reaching sequence, producing a data series of 1, 12, 3, 18, 2, 8. Thus, a “steeper” pattern on this measure (as in the first example) indicates a reaching sequence that is highly ordered based on object distance, while a “flatter” pattern (as in the second example) indicates a greater willingness to reach to distant objects early in the reaching sequence. In the discussion we consider the potential relationship between this pattern and representation of the space around the self.

**Fig 6:**
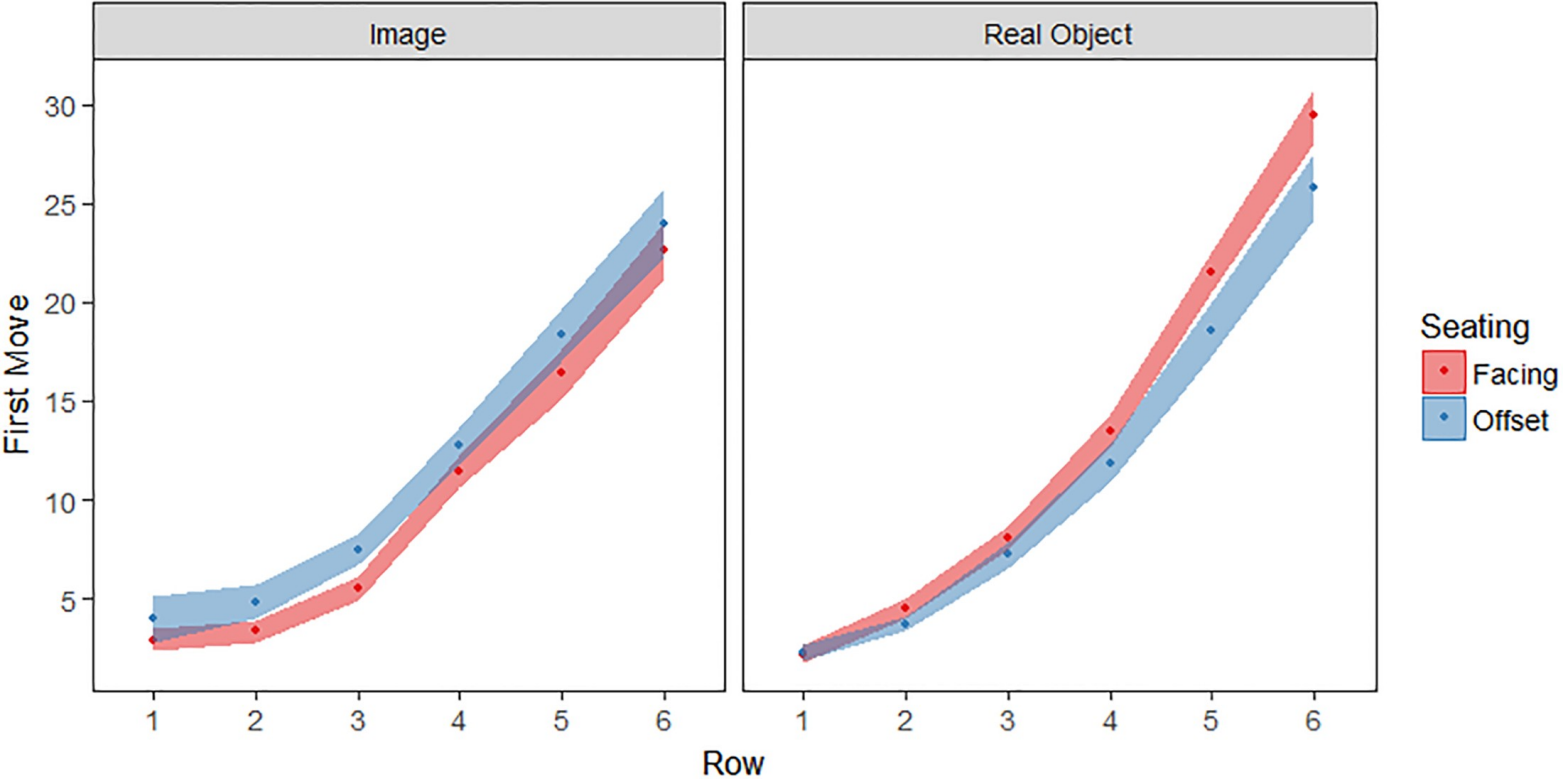
The number of moves (*M*±*SE*) preceding first entry into each row of real objects and images. Higher values indicate that a particular location was contacted for the first time at a later point in the reaching sequence.

A repeated-measures ANOVA was performed as before, with the number of moves before first entering each row as the dependent measure. There were main effects of row (*F*(5, 795) = 380.8, *p* < .001, η_p_^2^ = .71) and stimulus type (*F*(1, 159) = 4.9, *p* = .03, η_p_^2^ = .03), and a significant interaction between the two, (*F*(5, 795) = 4.6, *p* < .001, η_p_^2^ = .03) reflecting the fact that more distant real objects were reached for later than equivalently placed images (Fig 6). Most importantly, there was an interaction between seating and stimulus type (*F*(1, 159) = 7.7, *p* = .006, η_p_^2^ = .05) reflecting that the social effect of sitting position differed between the real object and image tasks. Follow-up ANOVAs revealed that the reaching sequence performed in the Object-Facing condition differed from that of the Object-Offset (*F*(1, 81) = 4.41, *p* = .04, η_p_^2^ = .05) and the Image-Facing conditions (*F*(1, 84) = 15.8, *p* < .001, η_p_^2^ = .16). Performance in the Image-Facing condition did not differ from that of the Image-Offset condition (*F*(1, 78) = 3.36, *p* = .07, η_p_^2^ = .04), nor did the Object-Offset and Image-Offset conditions differ from one another (*F*(1, 75) = .13, *p* = .72, η_p_^2^ = .002). This last comparison is especially noteworthy as it indicates that a performance difference the two offset conditions does not drive the seating by stimulus type interaction. In other words, the fact that the two offset conditions were not perfectly matched–specifically, the non-playing participant was seated in front of their own array in the real-offset condition but not the image-offset condition is not the source of the seating by stimulus type interaction. Rather, this interaction is due to performance differences in the facing condition.

## Discussion

The present work investigated the following specific question: When social phenomena are examined, are images good proxies for studying actions to real objects? The assumption that images and real objects are reasonably interchangeable underlies a great deal of work investigating social phenomena using on-screen stimuli [1–3,41,48–52]. Discrepant with this, we found that images and real objects elicited different action sequences despite being placed into similar spaces, and that a basic social manipulation affected reaching behaviour for real items but not images. This work is relevant for those interested in studying social phenomena in lab or workplace environments. It suggests limitations on the generalizability of behaviour directed towards objects versus images, consistent with the previous literature [12,13,15]. Moreover, the novel contribution of this work is to provide evidence that in addition to overall differences between the processing of real objects and images, the two types of stimuli may also be differently sensitive to social context.

In this work, we used a real-action paradigm to compare actions elicited by the two stimulus types. Our reasoning was that if social effects exist in such a complex and relatively uncontrolled task, we could feel confident that they could also take place outside of highly controlled laboratory environments. We found that the social manipulation (observer location) had subtly different effects on the actions performed for real or on-screen items. In the real-object context, facing another individual led participants to delay contacting new rows relative to the other social condition (i.e., the offset observer). This social effect was eliminated and trended towards reversing for images. This result indicates that social effects are reduced (and may possibly even be reversed) when participants “handle” images compared to real objects. This was true despite the fact that the Facing condition for images placed participants slightly closer together than the Facing condition for real items. This reduction in the social effect for images is in line with observations made within the virtual-tabletop literature; sensitivity to social norms when handling real items (e.g., one avoids taking items out of another person’s hands or competing for control over a particular location) may not be observed on virtual tabletops displaying images [26,27].

Previous work has shown that real objects and images elicit different patterns of action and are supported by partially dissociable neural circuitry [4,5,10,11,16,21,22]. Some have suggested that these differences are due to the fact that real objects afford reaching and handling while images do not, or do so to a lesser extent [11,12]. Despite the fact that both stimulus types were placed on a reachable, touchable surface, the real objects used in our task required a more difficult manipulative action (grasping and flipping) than did the images (tapping). This difference alone should predict differences in stimulus-directed actions, since reach-to-touch and reach-to-grasp actions elicit different patterns of neural activity, as do image- versus object-directed actions [21,22]. However, it was unknown how these differences at the neurological level might manifest in the context of a complex, multi-reach task. More importantly, we were interested in how differences between image- and object-directed actions would interact with the larger social context in which those actions were performed.

Social context aside, we found persistent differences between actions directed towards real objects and images in three domains: timing, error rate, and sequence. Thus, the two stimulus types were associated with two different action profiles. On the whole, when compared to image-directed reaching sequences, object-directed reaching sequences involved longer reach times, a “steeper” distribution was observed (that is, distant objects were contacted later in the reaching sequence), and fewer erroneous moves were made. When turn length data were corrected for accuracy using efficiency scores, object-directed actions were found to be more efficient than image-directed actions. Thus, the different patterns of action seen for the two stimulus types do not constitute a speed-accuracy trade-off.

Classic work has demonstrated that the reaction time taken to initiate an action increases as a function of the difficulty of the planned action [53]. Though we calculated turn length as the time taken from line touch to item touch (explicitly excluding the additional time required to rotate the real items), the fact that real items require this rotation could still manifest in longer turn durations, and this could contribute to the overall differences in the timing structure of the two conditions.

Note as well that our pattern of results can also be interpreted with reference to recent findings concerning social effects on the representation of near space. Peripersonal space (PPS) refers to interconnected frontal and parietal brain regions that represent items and locations near to the body [54]. Recent work suggests that PPS size “shrinks” when facing a passive individual but not a mannequin [39]. This PPS remapping is also sensitive to the relationship between the two individuals [39,40,55]. It remains an open question whether PPS plasticity has a role in shaping image- and real object-directed behaviour. However, on-screen images do not activate PPS neurons in the same manner as real stimuli, suggesting that differences may be expected [6,7]. This is consistent with our finding that, when handling real items, facing another individual results in “steeper” reaching sequences (i.e. delayed reaching to items at a particular distance) than the offset condition involving real objects *and* the facing condition when handling images. While we have no direct evidence that reaching sequence measured in this way maps onto the size of an individual’s PPS, these two phenomena seem to be compatible. Specifically, a lack of interaction with more distant objects (as shown by a “steep” reaching sequence) occurs in the same condition (Object-Facing) in which we would expect participants to show the smallest PPS. The present work does not directly test peripersonal space and manipulation difficulty as mechanisms by which real objects might be more sensitive to social manipulations; however these provide interesting avenues for further research.

This work provides helpful context for existing work research that finds that social factors such as requests for items or interpersonal relationships can shape action kinematics for object-directed actions [28,32]. Rather than examining the trajectory of individual reach and grasp actions, the present task examined larger features (e.g. sequence, location of errors) of a series of reaches with self-selected (rather than experimenter-selected) endpoints. The current data demonstrate that, in addition to modifying the structure of individual actions (as shown by Becchio and colleagues), social context can also alter object-directed reaching at the level of the larger reaching sequence. However, this was not the case for image-directed reaching sequences. It would be interesting for future work to examine whether image-directed reaches are also less sensitive to social context at the kinematic level.

This work also connects to an emerging literature that finds that social stimuli often elicit different behaviour than non-social stimuli, despite superficial similarities. For example, participants look less often at another live person in a waiting room as compared to a video of the same individual [56]. Similarly, the impact of a gaze cue differs when it is presented during a live social interaction as compared to when it is presented via an on-screen picture of a face [57]. In such experiments, two factors are confounded: stimulus type (objects versus images) and animacy (one-way stimuli that cannot “look back” at the participant versus two-way stimuli that can do so). The present work focuses only on the first factor–the nature of the stimulus–while controlling for stimulus animacy. Thus, one can consider whether real and non-real inanimate items are intrinsically differentially sensitive to social manipulations. The data suggests that they are. The present work is complemented by recent work examining looking behaviour directed towards virtual stimuli (videos of faces) in which participants believed their performance would (two-way) or would not (one-way) be seen by the subjects of the videos [58]. In a certain sense, this can be considered as the inverse of the present work: an examination of the effect of stimulus animacy while controlling for stimulus type. Further work is needed to continue to disentangle these two factors as they drive human behaviour.

Many model tasks are being developed that measure participants’ responses to images after a social manipulation. Tasks that have sought to examine social influences in this manner include measures of reaction time [3,59], object tracking [1], timing judgments [49], spatial attention [48,60], memory [50,51], and visual search performance [2], among numerous other examples. In general the evidence indicates that the social effects in these tasks are statistically small [61]. Our data suggest that by using such paradigms, authors risk underestimating the contributions of social factors to object-related cognition, or possibly erroneously concluding that certain phenomena are not sensitive to social factors when this holds true for images but not real objects. Thus the results of the present study provide evidence that researchers should exercise caution in generalizing social effects measured with images to more naturalistic situations involving real objects. This is prudent not only for researchers in the lab who use images that serve as proxies for real items (e.g., pictures of people, graspable objects, food, fear inducing stimuli, etc.) but also for those in industry seeking to relate social behaviours in virtual, mixed and/or augmented realities to social effects in real life.
